# PCSK9 inhibitors in a renal transplant patient complicated with hepatitis B: A case report and literature review

**DOI:** 10.3389/fcvm.2022.937474

**Published:** 2022-11-07

**Authors:** Pinchao Lv, Yuxi Li, Lin Wu, Haoyu Weng, Ming Chen, Wenhui Ding, Jianping Li

**Affiliations:** Department of Cardiology, Peking University First Hospital, Peking University, Beijing, China

**Keywords:** dyslipidemia, renal transplantation, viral hepatitis, PCSK9i, coronary heart disease

## Abstract

Lipid metabolism disorders are recognized to be one of the most frequent complications of renal transplantation, while dyslipidemia and chronic kidney disease (CKD) are strong risk factors for arteriosclerotic cardiovascular disease (ASCVD). Proprotein convertase subtilisin/kexin type 9 inhibitors (PCSK9i) are novel lipid-lowering drugs, the safety and efficacy of which are yet to be confirmed in transplanted patients. There have been several small-sample studies using PCSK9i in patients after heart transplantation, while fewer cases use PCSK9i after kidney transplantation. We report a case of a renal transplant recipient complicated with hepatitis B treated with PCSK9i, which achieved a remarkable lipid-lowering efficacy, and no significant adverse effects were found during the follow-up.

## Introduction

Cardiovascular disease (CVD) is a major cause of mortality in renal transplant recipients. Many cardiovascular risk factors in this population, such as dyslipidemia, immunosuppressive agents, anemia, renal insufficiency, etc., increase the prevalence of ASCVD (1). Therefore, early intervention for these risk factors should be undertaken to improve the long-term outcome and reduce CVD death (2). The long-term inflammatory status of chronic hepatitis B patients may also increase the CVD risk by changing the lipid profile directly or indirectly (3). At present, there has been no case of kidney transplantation recipients complicated with hepatitis B treated with PCSK9i reported in the literature. In our case, a kidney transplant recipient with hepatitis B was admitted to the hospital due to acute coronary syndrome and heart failure; we discussed the challenges in drug therapy for secondary prevention of coronary heart disease with a focus on the patient’s lipid management.

## Case presentation

A 53-year-old female was diagnosed with glomerulonephritis at 22 and received kidney transplantation at 33. Her renal function was maintained with an immunosuppressant regimen of cyclosporine, mycophenolic acid, and prednisone. The patient was complicated with chronic viral hepatitis B, CKD2, hypertension, and obsolete cerebral infarction. She was admitted to our hospital in November 2020 due to “chest tightness for half a year, bilateral lower extremity edema, and paroxysmal nocturnal dyspnea for more than 10 days.”

**FIGURE 1:**
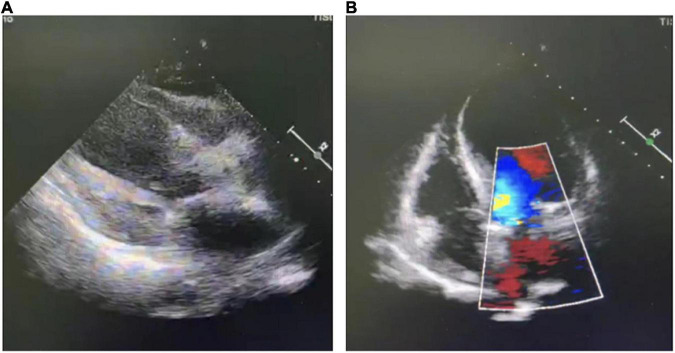
Ultrasonic echocardiogram. **(A)** Parasternal long axis view; **(B)** apical four chambers view.

**FIGURE 2:**
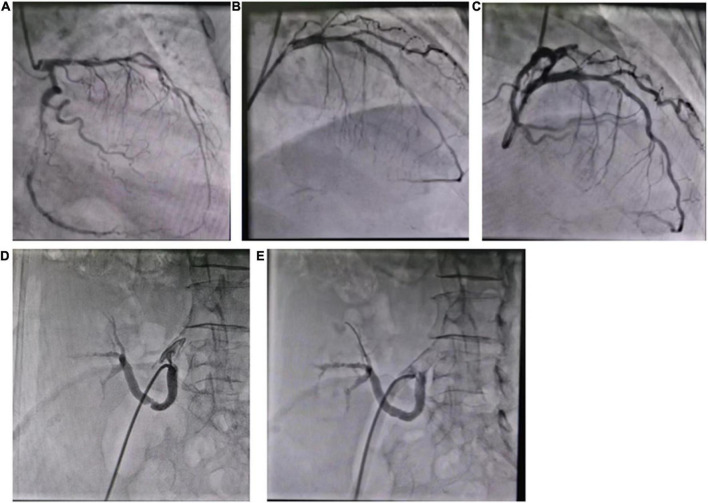
**(A)** Coronary angiography showed triple-vessel disease; **(B)** two stents were implanted in LAD; **(C)** two stents were implanted in LCX; **(D)** the renal artery angiography; **(E)** one stent was implanted in the renal artery.

**FIGURE 3:**
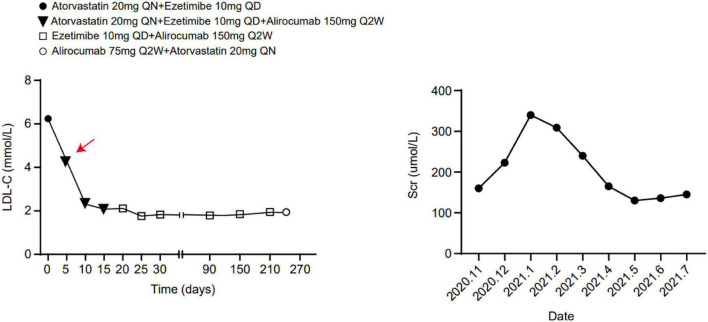
The lipid level and serum creatinine during hospitalization and follow-up.

The patient developed exertional chest tightness 6 months earlier, which was progressively worsening with resting angina and the symptoms of heart failure of paroxysmal nocturnal dyspnea, lower extremity edema, and oliguria 1 month earlier. Her edema was relieved after the symptomatic treatment of diuresis, coronary vasodilatation, and albumin infusion in other hospitals. The physical examination showed stable vitals, mild anemia appearance, no jugular irritation, normal regular heart rhythm and normal heart sounds, and bilateral lower extremity edema; the rest of her examination was unremarkable. Laboratory results showed white blood cell (WBC) 4.60 × 10^9^/L, hemoglobin 73 g/L, platelet 225 × 10^9^/L, erythrocyte sedimentation rate (ESR) 52 mm/h, hypersensitive C-reactive protein (hs-CRP) 2.02 mg/L, normal liver enzymes, albumin 32.0 g/L, serum creatinine (Scr) 165.75 mmol/L, estimate glomerular filtration rate (eGFR) 30 ml/min/1.73 m^2^, K^+^ 4.37 mmol/L, creatine kinase (CK) 102 IU/L, creatine kinase isoenzyme (CK-MB) 1.4 ng/ml, troponin I (cTnI) 0.617 ng/ml, BNP 2841 pg/ml, total cholesterol (TC) 9.15 mmol/L, low-density lipoprotein (LDL-C) 6.22 mmol/L, positive for HBsAg, HBeAg and HBeAb, HBV-DNA 987000.00 IU/ml, thyroid stimulating hormone (TSH) 12.51 uIU/ml, normal HbA1C, and blood concentration of cyclosporine A 156.50 ng/ml. The electrocardiogram (ECG) showed reduced R wave and T wave inversion in inferior and anteroseptal leads. Ultrasonic echocardiogram showed segmental left ventricular (LV) wall motion abnormalities (LV apex, interventricular septum), left atrial enlargement, thickening of the left ventricular wall and interventricular septal base, LVEF 52.7%, mitral valve (after leaves roots) calcification, mitral and aortic valve mild reflux, aortic sclerosis/calcification, and a small amount of pericardial effusion (Figure 1). Coronary angiography after the improvement of heart failure showed triple-vessel disease (proximal left anterior descending artery (LAD) 90% stenosis, middle LAD 85% stenosis, second diagonal branch 80% stenosis, distal left circumflex artery (LCX) 95% stenosis, and middle right coronary artery (RCA) 50% stenosis) (Figure 2A); two stents were implanted in each of the LAD and LCX, and the second diagonal branch underwent balloon dilation (Figures 2B,C and Supplementary material). Meanwhile, 80% stenosis in the renal graft artery was revealed during angiography. OCT (optical coherence tomography) showed that the renal artery area was narrowed by 50%. Afterward, the renal dynamic imaging showed decreased blood perfusion and impaired function of the transplanted kidney, thus one stent was implanted in the transplanted kidney artery (Figures 2D,E and Supplementary material). After interventional therapy, the patient developed contrast nephropathy (Scr 160→223 umol/l), urine output progressively decreased, and there was poor treatment effect for heart failure, which led to hemodialysis. Secondary prevention therapy for CHD included: aspirin 100 mg QD, ticagrelor 90 mg BID, carvedilol 25 mg BID, sacubitril/valsartan (stopped due to contrast nephropathy later), atorvastatin 20 mg QN (previous history of long-term use), and ezetimibe 10 mg QD for lipid-lowering therapy. Suboptimal LDL-c made us add the PCSK9i, alirocumab 150 mg Q2W. During hospitalization rhabdomyolysis occurred, so atorvastatin was stopped with the maintenance treatment of alirocumab + ezetimibe. Antiviral therapy with entecavir was directed by the infective consultation. During the 8-month follow-up, lipid profile and renal function were regularly checked (renal function was checked every 2 weeks, and lipid was checked every 2 months), and no cardiovascular event occurred. Regular dialysis was performed three times a week, urine output gradually recovered, and Scr recovered to the baseline level (140 umol/L). The patient was independent of dialysis after about 6 months with relatively stable clinical heart and kidney function. Given the high cost of alirocumab, the patient reduced the dose of the alirocumab and reinitiated atorvastatin on her own during the follow-up. Surprisingly, the patient did not have muscle pain during the re-administration of statins but the lipid profile had a trend of increasing. Recent LVEF was 56.7% by ultrasonic echocardiogram and the lipid levels improved (Figure 3). The tumor markers related to hepatic cancer were all in the normal range, and the abdominal CT did not reveal any signs of a tumor.

## Discussion

Lipid metabolism disorders are one of the most common complications after kidney transplantation, usually occurring 3–6 months after transplantation and lasting for more than 10 years (4). According to the European Cardiology Guidelines for Lipid Management 2019 (5), the management of dyslipidemia in transplant recipients is comparable to what is recommended for patients at high or very high ASCVD risk: an LDL-C reduction of ≥50% from baseline and an LDL-C target of <1.4 mmol/L (<55 mg/dL) are recommended (IA). Lower lipid levels reduce the risk of cardiovascular death, stroke, myocardial infarction, etc. The guidelines recommend statins as the first line of lipid-lowering therapy, in combination with ezetimibe or PCSK9i if necessary. Kidney transplant patients often show decreased tolerance to statins for a variety of reasons, so they need to combine with other drugs such as the new lipid-lowering drug PCSK9i. PCSK9 is a serine protease produced by the liver, which can degrade low-density lipoprotein receptors on hepatocytes and affect the transport of LDL-C into hepatocytes. Meanwhile, PCSK9 protein may contribute to the instability of coronary plaque through LDL-C oxidation and platelet aggregation, which may play a role in the development of CVD (6). Thus, PCSK9 inhibitors can play a protective role in CVD by reducing LDL-C concentration, antioxidant, and other effects and reducing the risk of cardiovascular events (7, 8). However, as a novel lipid-lowering drug, studies evaluating the efficacy and safety of PCSK9i therapy in reducing lipid and preventing cardiovascular outcomes, such as the FOURIER study and ODYSSEY Outcome study, have typically excluded patients with significantly impaired renal function, though the criteria used for exclusion are varied. Recently a *post-hoc* analysis of the ODYSSEY study showed that, in CKD3 patients (eGFR 30–59 ml/min/1.73 m^2^), LDL-C decreased by 46.1–62.2% after 24-weeks follow-up treated with PCSK9i. The lipid-lowering effect was independent of basic renal function; there were no significant differences in the rates of adverse effects between normal renal function and renal insufficiency groups (9, 10). Comparable results were obtained in the FOURIER study (excluded CKD patients of eGFR < 20 ml/min/1.73 m^2^), in which the reduction in cardiovascular death, myocardial infarction, and stroke were greater in patients with advanced renal insufficiency (11). However, the use of PCSK9i in transplant patients has been limited. At present, there are some case reports on the use of PCSK9i in patients after heart transplantation; the efficacy is roughly the same with the maximum reduction of LDL-C up to 4.4 mmol/L. Long-term clinical safety has yet to be confirmed. Some cases reported that patients after heart transplantation treated with PCSK9i had no serious adverse reactions during the follow-up period (7, 12). In another study of 65 patients taking PCSK9i after heart transplantation, four cases withdrew from the study due to adverse reactions: increased CK, severe abdominal pain, headache, shortness of breath, and joint pain (13). Nevertheless, there are few experiences of PCSK9i therapy in kidney transplant patients. In a case series involving 12 transplant patients, including one kidney transplant patient, the use of PCSK9i was shown to be safe and effective after 6 months of follow-up (14). Our patient received kidney transplantation 20 years ago and was admitted with ACS this time, she took statin before with a poor lipid-lowering effect so that was added with ezetimibe and PCSK9i to reach the lipid goal. After 8 months of follow-up, the patient’s LDL-C level went down significantly and was stable, consistent with the literature. No serious adverse events occurred during the follow-up period.

Another aspect worth discussing is the interaction between drugs, especially lipid-lowering drugs, and immunosuppressive agents in kidney transplant recipients, which needs to be closely monitored due to the long-term use of immunosuppressants. Different immunosuppressive agents affect blood lipid levels differently. For example, some immunosuppressants may affect lipoprotein metabolism through lipoprotein lipase inhibition and insulin resistance (15). The other immunosuppressants (e.g., cyclosporine) may increase serum PCSK9 levels. In contrast, mycophenolic acid and azathioprine have few effects on blood lipids. Animal experiments found an elevated plasma level of LDL-c and PCSK9 in LDL-c receptor knockout mice treated with cyclosporine, suggesting that LDL-c receptor-mediated lipoprotein removal has a protective effect on cyclosporine related hyperlipidemia, which means using PCSK9i can enhance the removal of LDL-c and reduce the cyclosporine mediated hyperlipidemia (16). In clinical studies, it was also found that the use of everolimus in certain genotypes and female patients was correlated with the increased concentration of PCSK9, suggesting the increased concentration of PCSK9 caused by immunosuppressive agents was the cause of lipid metabolism disorder (17). However, PCSK9i did not affect the p-glycoprotein pathway that metabolized statins and common immunosuppressant, CYP450, OATP1B1, and BRCP pathways. As a result, PCSK9i rarely interacts with most immunosuppressants in post-transplant patients (15). The combination use of immunosuppressants and PCSK9i has been reported in patients after heart transplantation, confirming its safety and good tolerance (18–20). However, another kidney transplant patient who received alirocumab in combination with everolimus for 2 years developed severe lung infections twice during the treatment period. After the replacement of everolimus with azathioprine, no recurrence of pneumonia was found (21). In addition to the relationship between PCSK9i and immunosuppressants, the potential drug interactions between statins and immunosuppressants must also be considered, especially with cyclosporine, which is metabolized through CYP3A4 and may increase the blood levels of all statins’ exposure and the risk of myopathy (1). The effectiveness of statin in renal transplant patients was uncertain; the guideline recommended statin to be the first-line agent in transplant patients but initiation should be at low doses with very careful observation (5). However, everolimus, of the same classification of calcineurin inhibitor (CNI) as cyclosporine, rarely has drug-drug interaction with atorvastatin (1). Therefore, choosing the appropriate combination of statin and immunosuppressant is essential in post-transplant patients. As for the interaction between ezetimibe and immunosuppressants, it has been confirmed by previous studies that the combination of the two drugs is relatively safe and effective (22). In statin-intolerant patients or those still with dyslipidemia despite maximally tolerated statin treatment, alternative or additional therapy with ezetimibe may be considered (5). Notably, cyclosporine can induce a 2–12 fold increase in the ezetimibe level. Our patient was administrated with cyclosporine, mycophenolic acid, and prednisone, and during the combined use of PCSK9i, statin, and ezetimibe, the cyclosporine plasma concentration was monitored to guide the drug dosage, atorvastatin was halted due to the rhabdomyolysis during the hospitalization. The patient reinitiated the statin and reduced the dosage of PCSK9i for financial reasons. Fortunately, no serious drug interaction was found during the follow-up.

It is also notable that the patient in this case also had chronic hepatitis B. It is known that lipid profiles in chronic hepatitis B patients showed higher levels of TG and LDL-C, and lower levels of HDL-C compared with healthy control groups (3). PCSK9 concentration was correlated with liver cancer in the course of chronic hepatitis. It has been found that liver cancer can cause paraneoplastic hyperlipidemia by inhibiting lipoprotein receptors in non-tumor tissues (23). Our patient had chronic hepatitis B, and although the liver function was normal, it showed an obvious lipid metabolism disorder. We also screened the patients for liver tumors, and the results were all normal. To the best of our knowledge, there is no study on the use of PCSK9i in patients with kidney transplantation complicated with hepatitis B, only one case reported the efficacy and safety of early use of PCSK9i in heart transplant recipients receiving hepatitis C virus-positive donors (24).

## Limitations

Due to distance from the patient, the data on cardiac function during follow-up was lacking. In addition, she reinitiated statin therapy by herself, which would increase the possibility of adverse events and mislead us about the effectiveness judgment of lipid-lowering drugs.

## Summary

We report the first case of the efficacy and safety of PCSK9i in a renal transplantation patient with hepatitis B. There is a high prevalence of lipid metabolism disorders in renal transplantation and chronic viral hepatitis B population, and due to factors such as the complexity of medication and the underlying disease state, it is challenging to manage lipids in these populations. At present, the new lipid-lowering drug PCSK9i has only been proven to be effective and safe in case reports. Therefore, it is necessary to carry out a larger study to confirm the effectiveness and safety of PCSK9i in a broader population.

## Data availability statement

The original contributions presented in this study are included in the article/Supplementary material, further inquiries can be directed to the corresponding author.

## Ethics statement

The studies involving human participants were reviewed and approved by the Biomedical Research Ethics Committee, Peking University First Hospital. The patients/participants provided their written informed consent to participate in this study. Written informed consent was obtained from the individual(s) for the publication of any potentially identifiable images or data included in this article.

## Author contributions

JL and LW were responsible for the conception and design of the study. PL and YL drafted the article. HW, MC, and WD were responsible for the patient’s diagnosis and treatment. All authors contributed to the article and approved the submitted version.
